# Body evaluation in men: the role of body weight dissatisfaction in appearance evaluation, eating, and muscle dysmorphia psychopathology

**DOI:** 10.1186/s40337-024-01025-9

**Published:** 2024-05-21

**Authors:** David Dal Brun, Elena Pescarini, Sofia Calonaci, Elisa Bonello, Paolo Meneguzzo

**Affiliations:** 1 Department of Linguistic and Literary Studies, University of Padova, Padova, Italy; 2 Plastic Surgery Unit, San Bortolo Hospital, ULSS 8 Berica, Vicenza, Italy; 3 Department of Neuroscience, University of Padova, via Giustiniani 2, Padova, 35128 Italy; 4 Padova Neuroscience Center, University of Padova, Padova, Italy

**Keywords:** Body weight dissatisfaction, Male, Muscle dysmorphia, Eating disorder

## Abstract

**Background:**

Body image dissatisfaction is a significant concern among men, influencing appearance evaluation, eating behaviors, and muscle dysmorphia psychopathology. However, research on these correlations is notably deficient in men, largely because body image concerns are unevenly distributed between genders. Therefore, this study aims to assess the various dimensions of concerns about body image in men and explore their associations with characteristics such as sexual orientation.

**Methods:**

A cross-sectional study was conducted with 251 adult men from fitness centers in the Veneto Region, Italy. Participants completed self-report questionnaires, including the Eating Attitudes Test (EAT-26), the Appearance Schemas Inventory-Revised (ASI-R), and the Muscle Dysmorphic Disorder Inventory (MDDI). Body weight perception and dissatisfaction were evaluated using a Figure Rating Scale (FRS) with 12 male biometric silhouettes.

**Results:**

Participants were divided into three subgroups based on the experienced levels of body weight dissatisfaction: those who rated a smaller body (BWsmaller), those who rated a larger body (BWlarger) more highly relative to their own estimated body size, and those who reported implicit neutrality with their current weight (BWneu). BWsmaller participants reported higher levels of eating-related concern, while BWlarger participants exhibited higher levels of muscle dysmorphia. Additionally, the BWlarger group showed the highest degree of quantitative perceptual underestimation of their body weight. Sexual orientation was found to have an impact on body weight dissatisfaction, with bisexual men more likely to desire an increase in weight and gay men more likely to desire a decrease.

**Conclusions:**

Body weight dissatisfaction significantly impacts appearance evaluation, eating behaviors, and muscle dysmorphia psychopathology in males. Tailored interventions that consider individual differences can support the well-being of men. The study provides useful insight into male body image issues, warranting further exploration to inform effective interventions and promote positive body image and mental health in this population.

## Abstract (fr)

This study delved into how males feel about their bodies and the impact it has on their thoughts and behaviors. We surveyed adult males and found three groups based on body weight satisfaction: those who wanted to lose, gain, or maintain their weight. Men wanting to lose weight reported more concern about eating, while those wanting to gain weight showed higher levels of muscle dysmorphia, a desire for more muscle mass. Interestingly, sexual orientation influenced these feelings, with bisexual men wanting weight gain and gay men preferring weight loss. This study highlights the importance of tailored support for males based on their unique needs to improve their well-being.

## Introduction

Body image is a multifaceted concept that encompasses various elements, including cognition, feelings, perceptions, beliefs, and behaviors [1, 2]. Dissatisfaction with one’s own body weight (BWD) and the resulting psychopathology are affected by distortions in perception and appreciation of one’s own body and the anxiety associated with body image [3]. BWD can manifest itself in several different ways, ranging from concerns about having an insufficiently muscular or small body to concerns that translate into an overestimation of one’s body weight and/or shape [4, 5]. In men, BWD has been associated with the desire to increase weight, higher levels of depression, and being part of sexual and gender minorities, while it seems to be not related to age [6–10]. Moreover, BWD appears to be unrelated to a specific weight or the presence of eating disorder psychopathology in both men and women, suggesting the potential presence of these concerns in various conditions, ranging from underweight to obesity [10]. Only in men has body dissatisfaction been related to drive to muscularity and exercise dependence, with a partial mediation of muscularity between body dissatisfaction and exercise dependence [11].

In exploration of the extremes of the BWD range, muscle dysmorphia (MD) is identified as a mental and behavioral condition in which people perceive themselves as inadequately muscular, despite possessing above-average muscularity [12]. This phenomenon has been proposed to serve as a male counterpart to a more female-centered construct of anorexia nervosa, with a higher prevalence among men and a distinctive focus on muscularity rather than thinness [13]. Studies consistently associate MD with eating disorder symptoms and neurocognitive deficits similar to those observed in anorexia nervosa [14]. Furthermore, MD individuals exhibit unique psychopathological traits, psychosocial impairment, and increased risk of suicide risk compared to those with other forms of body dysmorphic disorder, highlighting the need to integrate MD into eating disorder classification schemes [13, 15]. Doing so would help alleviate the female-centric bias inherent in current classifications and highlight the growing problem of male body image concerns [16, 17].

Clinical and research evidence suggests high crossover rates among different diagnoses of eating disorders, especially in men [5, 18], with a specific suggestion for the inclusion of MD in the spectrum of ED, as well as for the presence of high levels of drive towards a lean and muscular body in different age groups [13, 19]. This approach may help reduce the number of men diagnosed with subthreshold ED [5]. However, the psychopathology of ED is often underdiagnosed in men [20], and MD has been primarily studied in male athletes as opposed to the general population [21], which has helped reinforce the stigmatizing notion that EDs are inherently female psychiatric conditions, while MD is a male disorder [22]. However, BWD transcends gender boundaries and is more closely related to other factors such as sexual orientation, age, sociocultural pressure, and interpersonal influences [3, 23–25].

Contrary to popular belief, the existing literature has pointed to similar levels of body dissatisfaction in men and women [26], with a recent trend towards an increased focus on muscularity and body appearance in men [27]. Current knowledge of this topic has shown how young men’s body dissatisfaction can be influenced by exposure to images of ideal male bodies, amidst a growing societal pressure on men’s appearance [28]. The primary concern of men is their muscularity, with approximately 50% of adult men reporting a desire to lose weight looking for lean bodies with muscles [13, 29, 30]. Thus, body composition, rather than thinness, plays a specific role, with higher (though not obese) body weight demonstrating a protective effect on male mental well-being. However, many studies have focused primarily on women or used assessments that may not resonate with men [31, 32], and a recent literature review suggests the need for new studies in this area [21].

Therefore, the main objective of this study is to evaluate the relationships between BWD, ED psychopathology, and muscle dysmorphia in male. Including different types of BWD allows for the evaluation and replication of the findings of the existing literature, facilitating direct comparisons between groups traditionally studied in isolation. Specifically, we seek to explore the psychopathological characteristics associated with different types of BWD (negative, neutral, or positive) and hypothesize that BWD might be related to specific psychopathology in male. Our goal is to develop a straightforward and helpful screening tool to improve the well-being of male.

## Methods

### Participants

Adult men (18 years of age or older) were invited to participate in a study aimed at investigating the relationship between physical training and body image. Participants were recruited through the mailing lists of various fitness centers in the Veneto Region (North East of Italy) between January and June 2023. All responders volunteered to participate and the data were collected anonymously through online surveys. To prevent multiple responses from the same IP addresses, the online survey was designed to reject multiple responses from the same address, and the IP addresses were kept hidden from the investigators. Only complete surveys were considered for the evaluation.

Written informed consent was obtained from each participant prior to completion of the survey. The study was approved by the local Ethics Committee and abided by the rules and guidelines set by the national legislation for anonymous surveys. It was conducted following the principles outlined in the Declaration of Helsinki, including its subsequent amendments.

### Measures

We collect demographic information from the participants, including age, race, education, height, and weight. To determine gender, participants were asked to self-identify as cisgender, transgender, or non-binary. Each participant also indicated sexual orientation by stating if they identified themselves as heterosexual, bisexual, gay, or asexual, as applied in previous studies [3, 33]. Specific items in the questionnaire were used to evaluate previous psychiatric diagnoses (‘Have you suffered in the past or do you currently suffer from an eating disorder?’ ‘Has a psychologist or psychiatrist ever told you that you have body dysmorphic disorder?’). Various psychological constructs were evaluated using specific self-report questionnaires.

The Eating Attitude Test (EAT-26) is a 26-item questionnaire that is used to identify concerns about eating disorders based on attitudes, feelings, and behaviors related to eating [34]. Items are assessed by means of a Likert scale of 6 points with a range score between 0 and 78. The clinical cut-off point is set at 22. In this study, the questionnaire demonstrated good reliability, with a Cronbach’s α of 0.786.

The Appearance Schemas Inventory–Revised (ASI-R) is a 20-item questionnaire rated on a 5-point Likert scale, ranging from ‘strongly disagree’ (1) to ‘strongly agree’ (5), designed to assess body image investment [35]. The questionnaire consists of two factors: self-evaluative salience (α = 0.890) and motivational salience (α = 0.840).

The Muscle Dysmorphic Disorder Inventory (MDDI) is a 13-item questionnaire rated on a 5-point Likert scale from 1 (never) to 5 (always), designed to assess cognitive, behavioral, and emotional features of muscle dysmorphia [36]. For this study, we used only the total score (α = 0.780).

To evaluate the perception and dissatisfaction in participants, we used a Figure Rating Scale (FRS) with 12 male biometric silhouettes, covering BMIs from 14 to 36 kg/m2, increasing by 2 kg/m2 from left to right, with a center of 25 kg/m2 [33, 37]. This specific FRS has been created using statistical body image of average shape variation in the male population that was learned from 3D body scans of an international project about the creation of a biometric FRS with more than 6000 people involved (of both genders) and already applied to different populations [37–39]. Therefore, the silhouettes were not specifically created to identify muscularity, but to identify average body composition. Body weight dissatisfaction (BWD) was assessed by subtracting the selected ideal figure from the estimated current figure, while perceptual distortion (PD) was determined by subtracting the current figure from the estimated current figure.

### Statistical analysis

Participants were divided into three subgroups depending on their BWD: men who rated smaller bodies (BWsmaller), men who rated larger bodies (BWlarger), and men who rated their current weight, showing implicit neutrality for their body weight (BWneu). The demographic and psychological characteristics of the participants were evaluated using different ANOVAs, with post hoc analyses corrected by the Bonferroni method. The data was verified for ANOVA assumptions: normal distribution, homogeneity of variances, and independence of the samples. For sexual orientation, we used the chi-square analysis to determine the distribution in the subgroups. A multinomial logistic regression with BWneu as a reference for the analysis was applied to evaluate the risk connected with the psychological and demographic data. In the regression, only variables with significant differences in the ANOVAs between the BWD groups were included as factors: BMI, BMI min, BMI max, EAT-26 total score, MDDI, ASI-SES, FRS-PD, and sexual orientation. The data was analyzed using SPSS Statistics Version 25.0 (IBM Corp, Armonk, NY, USA).

## Results

A total of 276 male participants volunteered to participate in the survey. Of them, 25 didn’t fulfill the entire questionnaires and then were excluded. The remaining 251 men reported a mean age of 26.72 ± 4.89 years, a BMI of 22.65 ± 2.54, and an average education duration of 10.91 ± 4.55 years. All participants identified as cisgender and white and none reported a history of psychiatric conditions and current or previous use of psychiatric medications.

Looking at the BWD subgroups, we found: BWsmaller, *N* = 97; BWlarger, *N* = 90; and BWneu, *N* = 64. Notably, BWneu reported the lowest scores on all questionnaires. Interestingly, no differences emerged for body appearance motivation salience, while the desire to increase or reduce body weight was equally linked to self-evaluative salience. Eating concerns were higher in men who wanted to lose weight, while MDDI scores were higher in people with a desire to increase body weight. Moreover, BWlarger reported the highest levels of underestimation of their body weight.

**Table 1:** Demographic and psychological comparisons between subgroups

	Total sample *N* = 251	BWsmaller *n* = 97	BWneu *n* = 64	BWlarger *n* = 90	F	*p* pη^2^	
Age, years	26.72 (4.89)	27.39 (4.76)	26.38 (4.93)	26.38 (4.93)	1.527	0.219 0.012	
Education, years	10.91 (4.55)	11.40 (4.76)	10.66 (4.45)	10.56 (4.39)	0.939	0.392 0.008	
BMI, kg/m^2^	22.65 (2.54)	23.83 (2.31)	22.77 (2.12)	21.29 (2.41)	28.680	< 0.001 0.188	BWlarger < BWneu (< 0.001) BWsmaller < BWneu (0.014) BWlarger < BWsmaller (< 0.001)
BMI max, kg/m^2^	24.18 (4.03)	27.00 (3.88)	23.95 (3.13)	21.30 (2.33)	73.948	< 0.001 0.374	BWlarger < BWsmaller (< 0.001) BWlarger < BWneu (< 0.001) BWneu < BWsmaller (< 0.001)
BMI min, kg/m^2^	20.64 (2.29)	21.98 (2.44)	20.70 (1.76)	19.17 (1.42)	48.272	< 0.001 0.280	BWlarger < BWsmaller (< 0.001) BWlarger < BWneu (< 0.001) BWneu < BWsmaller (< 0.001)
EAT-26	6.16 (5.99)	7.76 (7.27)	4.75 (2.24)	5.44 (5.96)	6.128	0.003 0.035	BWneu < BWsmaller (0.005) BWlarger < BWsmaller (0.022)
MDDI	26.17 (8.61)	24.75 (7.67)	20.52 (4.47)	31.71 (8.62)	45.877	< 0.001 0.270	BWneu < BWsmaller (0.001) BWneu < BWlarger (< 0.001) BWsmaller < BWlarger (< 0.001)
ASI-SES	3.07 (0.76)	3.18 (0.70)	2.61 (0.77)	3.27 (0.68)	17.750	< 0.001 0.125	BWneu < BWsmaller (< 0.001) BWneu < BWlarger (< 0.001)
ASI-MS	3.12 (0.62)	3.09 (0.61)	3.02 (0.72)	3.24 (0.55)	2.485	0.085 0.020	
FRS-PD	-0.43 (1.50)	0.26 (1.64)	-0.61 (1.49)	-1.04 (0.97)	21.187	< 0.001 0.147	BWneu < BWsmaller (< 0.001) BWlarger < BWsmaller (< 0.001)

Means and standard deviations are reported in the table. EAT-26: eating attitude test; MDDI_ muscle dysmorphic disorder inventory; ASI: appearance schemas inventory; SES: self-evaluative salience; MS: motivational salience; FRS: figure rating scale; PD: perceptual distortion; BW: body weight; neu: neutral

**Fig. 1:**
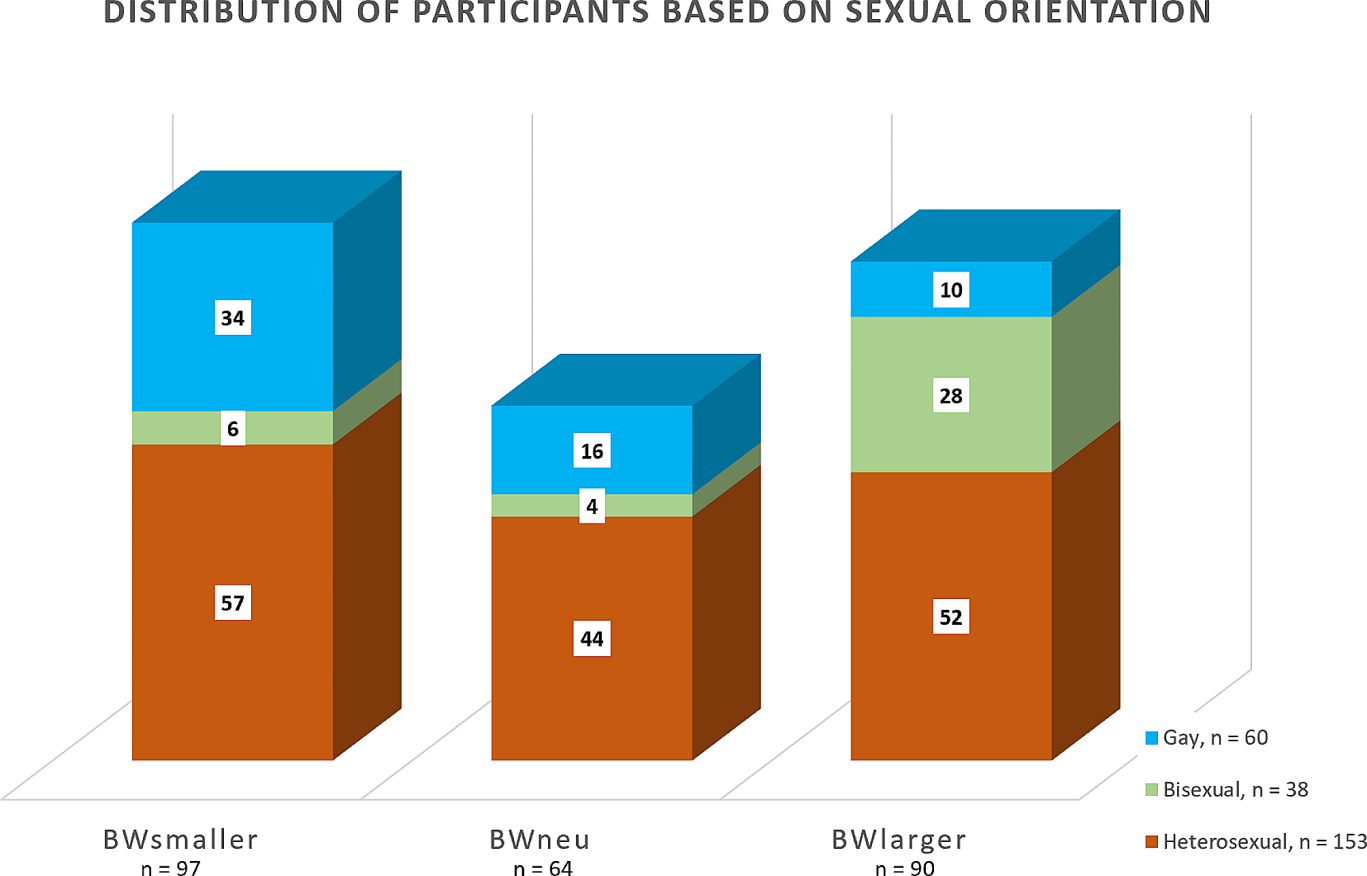
The figure presents the distribution of participants according to their sexual orientation (χ^2^ = 35.742, *p* < .001). For the heterosexual group (*N* = 153), the distribution is as follows: 37.2% of the sample was in the subgroup that reported a desire for a smaller body, 28.8% in the group with a neutral attitude through their body weight (BWneu), and 34.0% in the subgroup with a desire of a larger body. In the bisexual category (*N* = 38), the majority, 73.7%, were included in the BWlarger subgroup, while 15.8% experienced a desire to decrease and 10.5% had a neutral attitude. Among the gay participants (*N* = 60), 56.7% wanted to decrease their body weight, 26.7% had a attitude through their body weight, and 16.7% reported a desire of a larger body weight

Regarding sexual orientation, 153 men identified as heterosexual, 38 as bisexual, and 60 as gay. Significant differences were observed according to sexual orientation and BWD (*p* < .001), with most bisexual men reporting a desire to increase their body weight, while the majority of gay men reported a desire to reduce it. See Fig. 1 for details.

Multinomial logistic regression, with BWneu as the reference group, revealed that higher muscle dysmorphia and self-evaluative salience scores were associated with increased odds of being part of the subgroup that desire a larger, as well as a smaller body weight, rather than being neutral. Additionally, BWD-PD resulted with opposite odds in the models, with higher odds for the smaller subgroup, and higher odds for the larger one, suggesting a possible specific effects in the desire of change the own body weight. Specific effects were linked to the weight history, with a higher maximum BMI related to higher odds for the desire to decrease weight, and the current BMI related to higher odds for the desire of a larger body. Finally, sexual orientation resulted with smaller odds looking at the predictor ability of being part of the subgroups with the desire of a smaller weight rather than the neutral one. See Table 2 for more details.

**Table 2:** Multinomial logistic regressions with BWneu as a comparison population

	*p*	Odds	95% CI
BWsmaller, χ^2^ (8,161) = 69.145, *p* < .001, R^2^ = 0.480			
BMI	0.359	0.857	0.616–1.192
BMI min	0.557	0.901	0.638–1.274
BMI MAX	< 0.001	1.684	1.259-2-253
EAT-26	0.938	1.006	0.875–1.156
MDDI	0.042	1.098	1.003–1.202
ASI-SES	< 0.001	3.331	1.692–6.558
FRS-PD	0.012	1.470	1.087–1.988
Sexual orientation	0.004	0.656	0.491–0.876
BWlarger, χ^2^ (8,154) = 157.55, *p* < .001, R^2^ = 0.870			
BMI	0.004	0.122	0.029–0.512
BMI min	0.426	1.674	0.470–5.962
BMI MAX	0.616	0.766	0.270–2.169
EAT-26	0.943	0.995	0.865–1.144
MDDI	< 0.001	1.972	1.382-2-814
ASI-SES	0.033	5.177	1.146–23.382
FRS-PD	0.009	0.256	0.093–0.707
Sexual orientation	0.785	0.921	0.508–1.667

EAT-26: eating attitude test; MDDI: muscle dysmorphic disorder inventory; ASI: appearance schemas inventory; SES: self-evaluative salience; MS: motivational salience; FRS: figure rating scale; PD: perceptual distortion; BWD: body weight dissatisfaction; BW: body weight; neu: neutral

## Discussion

The present study provides useful information on the associations between body image concerns and other psychological factors in male, shedding light on the complexities of body weight perception and its impact on well-being.

The results indicated that participants who reported implicit neutrality through their body weight reported the lowest scores on all questionnaires, indicating reduced psychopathological concerns compared to the other groups, according to preliminary data reported in the literature [40, 41]. In particular, there were no significant differences in the importance of motivation for body appearance between the groups, suggesting that the desire to change body weight may not be driven primarily driven by concerns about physical appearance or that it could be linked to a gender characteristic [35]. However, the importance of self-evaluation was equally related to both the desire to increase and decrease body weight, suggesting that individuals in both two groups may share similar self-evaluation patterns regarding their bodies [42]. More studies are needed to better understand the role of body schema inventory and body weight concerns in men, a topic that has been neglected for decades, although a growing body of studies has shown that male are not immune to body image issues [43].

Regarding specific psychopathological constructs, participants desiring to lose weight exhibited higher eating concerns, indicating a potential link between body weight dissatisfaction and attitudes and behaviors. On the other hand, individuals in the BWlarger group displayed higher levels of muscle dysmorphia, indicating a focus on muscularity and the presence of potential body image distortion in terms of muscular development. This is in line with the constructs evaluated, corroborating the complex relationships between body dissatisfaction and behaviors.

Moreover, the BWlarger group reported the highest levels of underestimation of their body weight, suggesting a discrepancy between their perceived and actual weight. This finding implies that people who seek to gain weight may have a distorted perception of their current body size, potentially impacted by the desire to achieve a particular body image ideal [44, 45]. This element is crucial in looking at possible clinical applications. In fact, altered perception of one’s own body has been associated with ED and psychological burden in different populations [3, 7, 39], suggesting that they might be considered as two elements that ought to be further studied.

In our analysis of the correlation between sexual orientation and BWD, we found significant differences based on sexual identity. Bisexual men were more likely to want an increase in body weight, whereas gay men were more likely to desire a decrease. This finding suggests that sexual orientation may play a role in shaping ideals of body weight and body image concerns among men, which warrants further investigation and expansion of preliminary data from the preliminary literature data [33, 46]. Indeed, several studies have reported differences between bisexual men and gay men concerning body image concerns. These differences may be attributed to the possible presence of internalized homonegativity, which drives bisexual men to desire a more muscular and masculine body [47–50].

Finally, multinomial logistic regression analysis suggests that concerns about muscularity and self-evaluation may be relevant factors in body weight dissatisfaction. These psychological elements should be taken seriously in men, while also keeping weight history into account [51]. This aspect is in line with the results in the ED field, which are quite exclusively about women, and could help to evaluate specific elements related to gender and sexual orientations.

### Clinical implications

The study findings hold promising clinical implications for body image interventions in men with body weight dissatisfaction. Tailored interventions, which address self-evaluative salience and muscle dysmorphia, can target specific concerns based on different subgroups (e.g., those desiring weight reduction or increase). Interventions should also address the discrepancy between perceived and actual body weight for those seeking to increase weight. Additionally, recognizing the influence of sexual orientation on ideals of body image and promoting body acceptance in an inclusive and diverse way can contribute to improve mental well-being. By incorporating these perspectives, clinicians can design effective interventions that foster positive body image and support the general well-being of men with body weight dissatisfaction.

### Limits and strengths

The results of the present study should be considered in light of several limitations. First, the study relied solely on self-report questionnaires, which may introduce potential biases in the responses of the participants. Furthermore, the cross-sectional approach employed in the study limits our ability to establish causal relationships between variables, as it only provides a snapshot of data at a single point in time. Furthermore, while the sample consisted exclusively of white cisgender men, which may be representative of the Italian population, it does not fully represent the diversity of the broader population. To enhance the generalizability of the results, future studies should aim to include a more diverse range of participants in terms of demographic characteristics.

We should also be aware of the strengths of the study, which involved a substantial number of men with various sexual orientations. Additionally, the study directly compared populations with opposing desires to change their body weights.

## Conclusion

In summary, the current study highlights the complex interaction between body weight dissatisfaction and various psychopathological constructs among adult men. The findings underscore the importance of considering individual differences and specific psychological factors when addressing concerns about body image in male populations. Understanding these associations can inform interventions and support customized approaches to promote positive body image and well-being among men. However, more research is needed to delve deeper the complex relationship between body weight dissatisfaction and psychological factors, considering additional sociocultural influences and potential long-term effects on mental health.

## Data Availability

No datasets were generated or analysed during the current study.

## Abbreviations

ASI: Appearance schemas inventory
BWD: Body Weight Dissatisfaction
BWsmaller: Body weight smaller
BWlarger: Body weight increase
BWneu: Body weight neutral
EAT-26: Eating attitude test
ED: Eating Disorder
FRS: Figure Rating Scale
MD: Muscle dysmorphia
MDDI: Muscle dysmorphic disorder inventory
MS: Motivational salience
PD: Perceptual distortion
SES: Self-evaluative salience
